# Supplementary material for: An open-source framework for large-scale, flexible evaluation of biomedical text mining systems
Source: J Biomed Discov Collab. 2008 Jan 29;3:1. doi: 10.1186/1747-5333-3-1 (PMC2276192; doi:10.1186/1747-5333-3-1)
Supplement: Additional file 1 — Evaluation platform source code. The additional file contains the software evaluation framework discussed in this paper. The most current release can be downloaded from SourceForge.net [1]. See the accompanying README file for instructions on installation and use. Also included in the distribution is a collection of UIMA wrappers for some commonly used BioNLP tools and annotated corpora. [file 1747-5333-3-1-S1.zip › bionlp_uima_repository-1.0.1/docs/api/src-html/edu/uchsc/ccp/uima/util/opendmap/DMAPJcasTokenizer.html]

```
001    package edu.uchsc.ccp.uima.util.opendmap;
002    
003    import java.util.ArrayList;
004    import java.util.Iterator;
005    import java.util.Vector;
006    
007    import org.apache.uima.cas.FSIndex;
008    import org.apache.uima.cas.FSIterator;
009    import org.apache.uima.jcas.JCas;
010    import org.apache.uima.jcas.JFSIndexRepository;
011    import org.apache.uima.jcas.cas.FSArray;
012    import org.apache.uima.jcas.cas.StringArray;
013    
014    import edu.uchsc.ccp.opendmap.DMAPToken;
015    import edu.uchsc.ccp.opendmap.DMAPTokenizer;
016    import edu.uchsc.ccp.opendmap.Parser;
017    import edu.uchsc.ccp.opendmap.Reference;
018    import edu.uchsc.ccp.uima.annotation.CCPTextAnnotation;
019    import edu.uchsc.ccp.uima.annotation.syntactic.CCPTokenAnnotation;
020    import edu.uchsc.ccp.uima.mention.CCPClassMention;
021    import edu.uchsc.ccp.uima.mention.CCPComplexSlotMention;
022    import edu.uchsc.ccp.uima.mention.CCPNonComplexSlotMention;
023    
024    /**
025     * Helper class to get all of the appropriate annotation structures
026     * out of the JCas and return them as DMAPToken structures
027     * for the OpenDMAP parser.
028     * 
029     * There are two types of annotations of interest at the moment:
030     * <ul>
031     * <li> CCPTokenAnnotation structures that correspond to individual words and pieces of punctuation
032     * <li> ClassMentionAnnotation structures that correspond to "named entities"
033     * </ul>
034     * 
035     * @author R. James Firby
036     */
037    public class DMAPJcasTokenizer extends DMAPTokenizer {
038            
039            /* The CAS iterator of the CCPTokenAnnotation structures */
040            private ArrayList<DMAPToken> tokens = new ArrayList<DMAPToken>();
041            
042            private Iterator<DMAPToken> tokenIterator = null;
043            
044            /**
045             * Create a new iterator over structures in the JCas.
046             * 
047             * @param parser The parser that will be using this tokenizer
048             * @param jcas The JCas holding the annotations to take as input
049             * @param annotationMap The map from JCas annotations to words and DMAP Protege Frames
050             * @param debug Print out additional debugging information if true 
051             */
052            public DMAPJcasTokenizer(Parser parser, JCas jcas, AnnotationMap annotationMap, boolean debug) {
053                    super();
054                    // Grab an iterator over the set of CCPTokenAnnotation structures in the JCas
055                    // Each of these corresponds roughly to a word
056                    JFSIndexRepository ir = jcas.getJFSIndexRepository();
057                    FSIndex index = ir.getAnnotationIndex(CCPTokenAnnotation.type);
058                    if (index != null) {
059                            FSIterator iterator = index.iterator();
060                            while (iterator.hasNext()) {
061                                    Object thing = iterator.next();
062                                    if (thing instanceof CCPTokenAnnotation) {
063                                            CCPTokenAnnotation jcasToken = (CCPTokenAnnotation) thing;
064                                            DMAPToken token = DMAPToken.newToken(parser, jcasToken.getCoveredText().toLowerCase(), jcasToken.getBegin(), jcasToken.getEnd());
065                                            token.setStart(jcasToken.getTokenNumber());
066                                            token.setEnd(jcasToken.getTokenNumber());
067                                            sortInNewToken(token);
068                                    }
069                            }
070                    }
071                    // Grab out any desired ClassMentionAnnotations.
072                    // These correspond to gene names, molecules and other named entities.
073                    if ((annotationMap != null) && (annotationMap.hasInputMentionsOfInterest())) {
074                            String docString = null;
075                            if (debug) {
076                                    System.out.println("\nPre-Annotated Mentions:");
077                                    docString = jcas.getDocumentText();
078                            }
079                            index = ir.getAnnotationIndex(CCPTextAnnotation.type);
080                            if (index != null) {
081                                    ArrayList<DMAPToken> semanticTokens = new ArrayList<DMAPToken>();
082                                    FSIterator iterator = index.iterator();
083                                    while (iterator.hasNext()) {
084                                            Object thing = iterator.next();
085                                            if (thing instanceof CCPTextAnnotation) {
086                                                    CCPTextAnnotation jcasToken = (CCPTextAnnotation) thing;
087                                                    DMAPToken token = buildDMAPToken(parser, jcasToken, annotationMap);
088                                                    if (token != null) {
089                                                            token.setSource(jcasToken);
090                                                            if (token != null) {
091                                                                    int start = 0;
092                                                                    for (int i=0; i<tokens.size(); i++) {
093                                                                            if (token.getCharacterStart() < tokens.get(i).getCharacterEnd()) {
094                                                                                    start = i;
095                                                                                    break;
096                                                                            }
097                                                                    }
098                                                                    int end = tokens.size()-1;
099                                                                    for (int i=0; i<tokens.size(); i++) {
100                                                                            if (token.getCharacterEnd() <= tokens.get(i).getCharacterStart()) {
101                                                                                    end = i - 1;
102                                                                                    break;
103                                                                            }
104                                                                    }
105                                                                    if ((start <= end) && (start >= 0)) {
106                                                                            token.setStart(start);
107                                                                            token.setEnd(end);
108                                                                            semanticTokens.add(token);
109                                                                    }
110                                                            }
111                                                            if (debug) {
112                                                                    AnnotationString desc = new AnnotationString(jcasToken, null, null, docString);
113                                                                    System.out.println(" " + token.getStart() + ".." + token.getEnd() + " " + desc.toString());
114                                                            }
115                                                    }
116                                            }
117                                    }
118                                    // Keep the list of tokens sorted by their end point
119                                    for (DMAPToken token: semanticTokens) {
120                                            sortInNewToken(token);
121                                    }
122                            }
123                    }
124    //              for (DMAPToken token: tokens) {
125    //                      System.out.println("Token: " + token.getStart() + "-" + token.getEnd() + "," + token.getCharacterStart()+ "-" + token.getCharacterEnd() + " '" + token.getItem().getText() + "'");
126    //              }
127                    tokenIterator = tokens.iterator();
128            }
129            
130            /**
131             * Build a DMAPToken representing a Protege Frame reference found in
132             * the JCas.
133             * 
134             * @param parser The parser that will be using the token
135             * @param jcasToken The JCas annotation that corresponds to this token
136             * @param annotationMap The configuration map from JCas annotations to DMAP frames
137             * @return
138             */
139            private DMAPToken buildDMAPToken(Parser parser, CCPTextAnnotation jcasToken, AnnotationMap annotationMap) {
140                    CCPClassMention mention = jcasToken.getClassMention();
141                    String type = mention.getMentionName();
142                    if (annotationMap.isInputMentionOfInterest(type)) {
143                            Vector<Object> slots = getMentionSlotValues(parser, mention, annotationMap);
144                            return DMAPToken.newToken(parser, annotationMap.getInputMentionReferenceType(type), slots, jcasToken.getBegin(), jcasToken.getEnd(), jcasToken);
145                    }
146                    return null;
147            }       
148            
149            /**
150             * Build a DMAP Reference for a JCas mention annotation.  The type of the Reference will
151             * corrspond to a Protege Frame and any mention slots will be turned into Reference slot/filler
152             * pairs.
153             * 
154             * @param parser The parser that will be using this reference
155             * @param mention The mention in the JCas this reference represents
156             * @param annotationMap The configuration map from JCas annotations to DMAP frames
157             * @return The reference
158             */
159            private Reference buildMentionReference(Parser parser, CCPClassMention mention, AnnotationMap annotationMap) {
160                    String type = mention.getMentionName();
161                    if (annotationMap.isInputMentionOfInterest(type)) {
162                            String dmapFrameName = annotationMap.getInputMentionReferenceType(type);
163                            Vector<Object> dmapSlots = getMentionSlotValues(parser, mention, annotationMap);
164                            return parser.newReference(dmapFrameName, dmapSlots);
165                    } else {
166                            return null;
167                    }
168            }
169    
170            /**
171             * Generate a list of DMAP slot/filler pairs for the slots in a
172             * JCas mention.
173             * 
174             * @param parser The parser that will be using the reference holding these slots
175             * @param mention The mention in the JCas that has the slots
176             * @param annotationMap The configuration map from JCas annotations to DMAP frames
177             * @return The list of slot/filler pairs
178             */
179            private Vector<Object> getMentionSlotValues(Parser parser, CCPClassMention mention, AnnotationMap annotationMap) {
180                    // Get the concept name
181                    String mentionName = mention.getMentionName();
182                    String dmapFrameName = annotationMap.getInputMentionReferenceType(mentionName);
183                    // Look at each slot
184                    Vector<Object> dmapSlots = new Vector<Object>();
185                    FSArray slots = mention.getSlotMentions();
186                    if (slots != null) {
187                            for (int i=0; i<slots.size(); i++) {
188                                    Object jcasSlotMention = slots.get(i);
189                                    if (jcasSlotMention instanceof CCPComplexSlotMention) {
190                                            // A slot that holds other mentions, need to create a nested Reference
191                                            CCPComplexSlotMention slot = (CCPComplexSlotMention) jcasSlotMention;
192                                            // Get the slot name and see if it should be included
193                                            String slotName = slot.getMentionName();
194                                            if ((slotName != null) && annotationMap.isInputMentionSlotIncluded(mentionName, slotName)) {
195                                                    // A requested slot, get the DMAP name
196                                                    String dmapSlotName = annotationMap.getInputMentionSlotReferenceType(mentionName, slotName);
197                                                    if (dmapSlotName != null) {
198                                                            FSArray slotClassMentions = slot.getClassMentions();
199                                                            for (int j=0; j<slotClassMentions.size(); j++) {
200                                                                    Object slotClassMention = slotClassMentions.get(j);
201                                                                    if (slotClassMention instanceof CCPClassMention) {
202                                                                            CCPClassMention classMention = (CCPClassMention) slotClassMention;
203                                                                            // Create a reference out of a slot filler class mention
204                                                                            Reference reference = buildMentionReference(parser, classMention, annotationMap);
205                                                                            // If we have a slot value, save it away
206                                                                            if (reference != null) {
207                                                                                    dmapSlots.add(parser.newReferenceSlot(dmapFrameName, dmapSlotName, reference));
208                                                                            }
209                                                                    }
210                                                            }
211                                                    }
212                                            }
213                                    } else {
214                                            // A simple text filler for the slot
215                                            CCPNonComplexSlotMention slot = (CCPNonComplexSlotMention) jcasSlotMention;
216                                            // Get the slot name and see if should be included
217                                            String slotName = slot.getMentionName();
218                                            if ((slotName != null) && annotationMap.isInputMentionSlotIncluded(mentionName, slotName)) {
219                                                    // A requested slot, get the DMAP name
220                                                    String dmapSlotName = annotationMap.getInputMentionSlotReferenceType(mentionName, slotName);
221                                                    if (dmapSlotName != null) {
222                                                            String slotValue = null;
223                                                            StringArray slotValues = slot.getSlotValues();
224                                                            // Just pull out the first value for now.  This should change in the future.
225                                                            if (slotValues.size() > 0) {
226                                                                    slotValue = slotValues.toArray()[0];
227                                                            }
228                                                            // If we have a slot value, save it away
229                                                            if (slotValue != null) {
230                                                                    dmapSlots.add(parser.newReferenceSlot(dmapFrameName, dmapSlotName, slotValue));
231                                                            }
232                                                    }
233                                            }
234                                    }
235                            }
236                    }
237                    return dmapSlots;               
238            }
239            
240            /**
241             * Check if there are any more tokens to process
242             */
243            @Override
244            public boolean hasNext() {
245                    // Are there any DefaultToken structures left
246                    if (tokenIterator == null) {
247                            return false;
248                    } else {
249                            return tokenIterator.hasNext();
250                    }
251            }
252    
253            /**
254             * Grab the next token and return it.  The tokens are in a
255             * list sorted by ending character (which roughly corresponds to
256             * input token order).
257             */
258            @Override
259            public DMAPToken next() {
260                    // Grab the next DefaultToken and turn it into a DMAP Token
261                    if ((tokenIterator != null) && (tokenIterator.hasNext())) {
262                            return tokenIterator.next();
263                    } else {
264                            return null;
265                    }
266            }
267            
268            /**
269             * Sort a new token into the list of tokens this tokenizer will
270             * return.
271             * 
272             * @param token The token to sort in
273             */
274            private void sortInNewToken(DMAPToken token) {
275                    boolean found = false;
276                    for (int i=0; i<tokens.size(); i++) {
277                            if (token.getCharacterEnd() < tokens.get(i).getCharacterEnd()) {
278                                    tokens.add(i, token);
279                                    found = true;
280                                    break;
281                            }
282                    }
283                    if (!found) tokens.add(token);
284            }
285            
286    }
```
